# A Novel Fertility Indicator Equation Using Estradiol Levels for Assessment of Phase of the Menstrual Cycle

**DOI:** 10.3390/medicina56110555

**Published:** 2020-10-22

**Authors:** Stephen J. Usala, A. Alexandre Trindade

**Affiliations:** 1 Department of Internal Medicine, Texas Tech University Health Sciences Center, Amarillo, TX 79106, USA; 2 Department of Mathematics and Statistics, Texas Tech University, Lubbock, TX 79409, USA; alex.trindade@ttu.edu

**Keywords:** fertile phase, estradiol levels, fertility awareness based method, FAM, natural family planning

## Abstract

*Background and Objectives*: Urinary hormone home monitoring assays are now available for fertility awareness methods (FAMs) of family planning, but lack sensitivity and precision in establishing the start of the fertile phase. We hypothesized that with a suitable algorithm, daily serum or blood estradiol (E2) levels could serve as a better analyte to determine the phase of the ovulatory cycle and the fertile start day (FSD). *Materials and Methods*: Published day-specific serum E2 levels, indexed to the serum luteinizing hormone (LH) peak, were analyzed from three independent laboratories for a threshold for a FSD. A fertility indicator quation (FIE) was discovered and tested with these data and a FSD was determined using the mean or median and variance ranges of the day-specific E2 data. *Results*: The considerable variance of day-specific serum E2 levels made an absolute serum E2 indicator for phase of cycle problematic. However, a FIE was discovered which maps the day-specific E2 levels of the ovulatory cycle enabling the fertile phase and transition to the luteal phase to be signaled. In this equation, FIE(**D**) is the value of FIE on day, **D**, of the cycle and has both a magnitude and sign. The magnitude of FIE(**D**) is the product of the normalized change in day-specific E2 levels over two consecutive intervals, (**D-2**, **D-1**) and (**D-1**, **D**), multiplied by 100, and is formulated as: (E2 (on **D-1**) − E2 (on **D-2**))/E2 (on **D-2**) × (E2(on **D**) − E2 (on **D-1**))/E2 (on **D-1**) × 100. The sign of FIE(**D**) is either + or − or ind (indeterminate) and is assigned on the basis of the direction of this product. Using a FIE threshold of ≥2.5 as the start of the fertile phase, the FSDs were Day −5 or Day −6, with FSD Day −4 for an outlier set of E2 levels. The maximum FIE value ranged 9.5–27.8 and occurred most often on Day −2. An inflection point with a large change in FIE magnitude and change in sign from + to − always occurred at Day 0 for all sets of day-specific E2 data signaling transition to the luteal phase. *Conclusions*: The fertility indicator equation, a product of two sequential normalized changes in serum E2 levels with a sign indicating confidence in direction of change, is powerful in identifying the fertile phase and subsequent transition to the postovulatory phase and may serve as a useful algorithm for FAMs of family planning.

## 1. Introduction

Fertility awareness methods (FAMs) of family planning have technically evolved with the objective metrics of urine luteinizing hormone (LH) and urine pregnanediol-3-glucuronide (P3G) to time ovulation and the luteal phase [1,2,3,4]. However, a sensitive and precise indicator and/or indicator algorithm is still lacking for the preovulatory fertile phase usually defined as the 6-day interval, Day −4 to Day +1 (Day 0 defined as LH peak) [5,6,7]. The fertile start day (FSD) can be problematic in assigning since there can be a fair amount of variance to the preovulatory phase [8]. This lack of certainty in the FSD increases the length of periodic abstinence required with present monitors and methods. For example, the Marquette method generally necessitates at least 11–12 days of abstinence which probably reduces more wide-spread use of this type of technology in FAMs [9,10]. Urinary estrone-3-glucuronide (E3G) is presently employed in devices such as the persona and with the Marquette method, but these devices can exhibit a relatively high pregnancy failure rate in FAMs of family planning [11,12]. It is unclear, given the lack of published, proprietary data on E3G in these devices, whether the unexpectedly high failure rate is due in part to lack of sensitivity and precision of the E3G assay or insufficient algorithms or both in identifying the FSD. Consequently, improved technology in determining the FSD during the preovulatory phase—either by assay or algorithm—could potentially reduce the anxiety of unintended pregnancy and the length of abstinence.

There appears to be more variance in day-specific urine E3G compared with day-specific serum E2 levels [12,13,14]. Serum E2 levels are presently the ‘gold standard’ to assess the state of the preovulatory phase; they closely reflect follicular development and the approach to ovulation [15]. Furthermore, fingerstick E2 measurements now have sensitivity and precision similar to serum E2 measurements [16,17]. A home fingerstick E2 monitor could be developed with sufficient market impetus.

We have explored the possible use of E2 as a metric to determine the FSD by examining day-specific serum estradiol levels throughout the menstrual cycle from three high performance assays and laboratories: Dighe et al. [18], Roos et al. [19], and Stricker et al. [20]. We found the present level of sensitivity and precision of serum E2 measurements makes a metric for FSD problematic. However, during our analysis we discovered a formulation—the fertility indicator equation—which enabled the FSD and transition to the luteal phase to be determined using day-to-day serum E2 levels in spite of the background of E2 variance.

## 2. Materials and Methods

### 2.1. Data Used: High-Resolution Serum Estradiol Levels during the Menstrual Cycle from Three Different Assays

Day-specific E2 levels throughout the ovulatory menstrual cycle were used in our study. Dighe et al., published high-resolution references ranges for serum E2 levels from 51 ovulating women with the Abbott AxSYM immunoassay platform [18]. They reported a functional sensitivity (i.e., the dose equivalent to a 20% between-run coefficient of variation (CV)) of 110 pmol/L (30 pg/mL). Only with E2 levels above 147 pmol/L (40 pg/mL) did they find CVs less than 20%. Roos et al., used the ADVIA Centaur XP Immunoassay System to measure day-specific E2 levels for 30 ovulating women, although for Days −12 to −16 there were only 1–5 subjects [19]. Stricker et al., established detailed reference values of serum E2 for 20 ovulating women using the Abbott ARCHITECT i2000 system [20]. Only Dighe et al. reported their own in-laboratory functional sensitivity measurements with replicate between run testing and found a difference with the manufacturer’s stated analytical sensitivity (73.4 pmol/L (20 pg/mL) vs. 110 pmol/L (30 pg/mL)).

### 2.2. The Delta Equation and Fertility Indicator Equation (FIE)

The novel FIE formulation, used to measure significant changes in the day-specific E2 levels from Dighe et al., Roos et al., and Stricker et al., was formulated as follows. For every day, **D**, of the cycle Delta function values are first calculated:

Delta (**D-1**) = (E2 level on Day, **D-1** − E2 level on Day, **D-2**)/(E2 level on **D-2**)

And

Delta (**D**) = (E2 level on Day, **D** − E2 level on Day, **D-1**)/(E2 level on **D-1**)

FIE on **D** is then calculated as the Delta product × 100:

(E2 on Day, **D-1** − E2 on, **D-2**)/E2 on **D-2**) × (E2 on **D** − E2 on **D-1**)/E2 on **D-1**) × 100

The sign of FIE is determined as follows:

+Delta (**D-1**) *×* +Delta (**D**) = +FIE

−Delta (**D-1**) *×* −Delta(**D**) = −FIE

However, they are indeterminate (‘ind’) if the product is (+) (-), (-) (+); that is—there is uncertainty in the direction of change.

**Figure 1:**
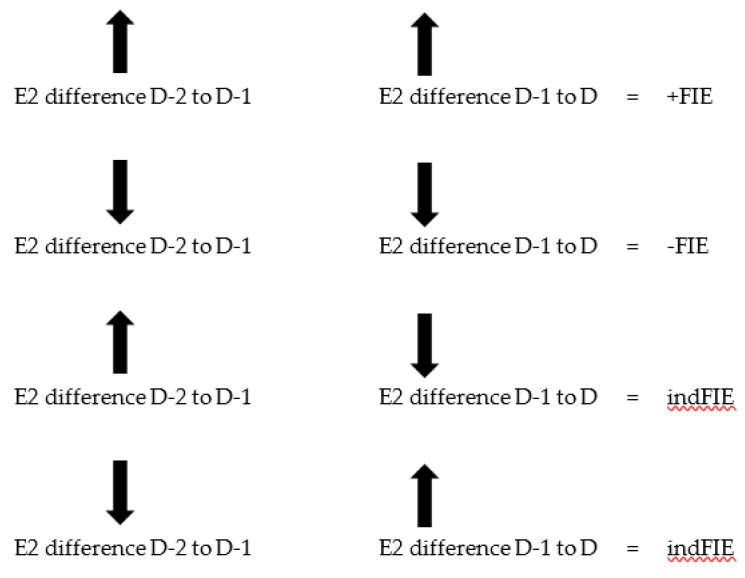
Sign determination for FIE (**D**) with magnitude formulation. The rules for determining the sign of FIE on day, **D**, throughout a cycle are shown. Up arrow is +, down arrow is −. The arrows indicate the possible sign combinations for the differences in the serum E2 levels for intervals, **D-2** to **D-1,** and **D-1** to **D**. The resulting signs for FIE can be +, −, or ind (indeterminate). The delta values for **D-1** and **D** are the normalized differences: Delta (**D-1**) = (E2 on **D-1** − E2 on **D-2**)/E2 on **D-2** and Delta(**D**) = (E2 on **D** − E2 on **D-1**)/E2 on **D-1**. The magnitude of FIE is the magnitude of the product: Delta(**D**) x Delta(**D-1**) × 100.

This formulation is further explicated in Figure 1. Calculations and graphing for delta (D) and FIE (D) were performed with Microsoft Excel and GraphPad Prism version 8 for Windows, GraphPad software, San Diego, CA, USA (www.graphpad.com).

## 3. Results

### 3.1. Analysis of Day-Specific Serum Estradiol Levels from Three Different Laboratories

Attention was first directed at serum E2 levels (rather than rate of changes) to determine if a threshold existed with commercial analyzers to establish the FSD for FAMs. If an E2 threshold existed, this would have relevance towards development of future home-based E2 monitors.

**Figure 2:**
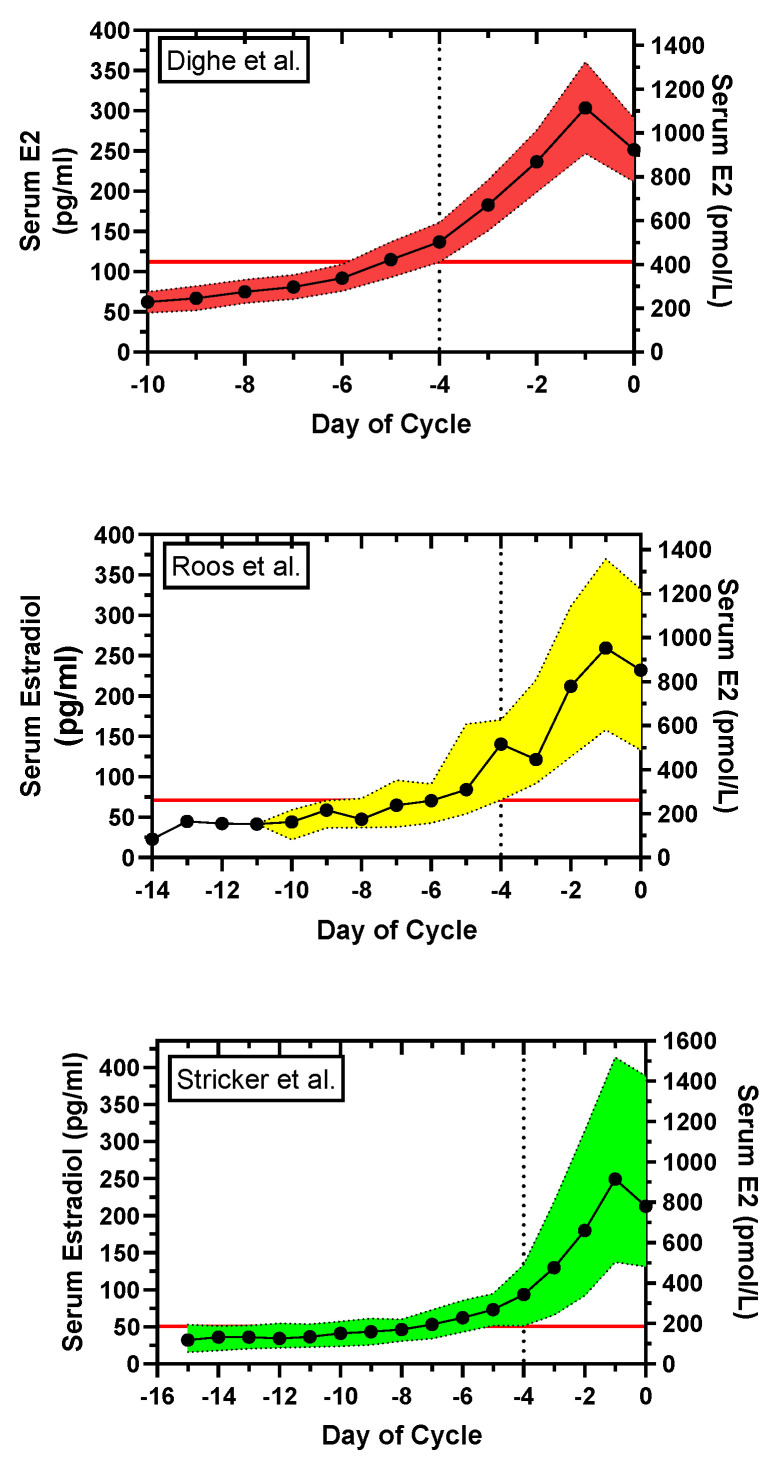
Plot of day-specific serum E2 levels, indexed to Day 0, day of serum luteinizing hormone (LH) peak, from three assays: Dighe et al. [18], Roos et al. [19], and Stricker et al. [20]. The dotted vertical line is at Day −4; the horizontal solid red line is at the lower limits (lower 99% CI, 10th percentile (PCTL), 5th PCTL) of the E2 levels. **Top**: The day-specific mean and 99% confidence interval (CI) limits for E2 are shown for Dighe et al. The lower 99% CI limit was 411 pmol/L (112 pg/mL) at Day −4. The interval, Day −4 to Day +1, is taken as the period of potential fertility [5,6,7]. The fertile start day (FSD), the day to start abstinence to avoid pregnancy with a fertility awareness method (FAM) of family planning, is determined by the lower limit of E2 at Day −4. To include the upper as well as the lower 99% limits of the cohort (red region), abstinence should begin on ~Day −6. **Middle**: The day-specific median and 10–90th PCTLs for E2 are shown for Roos et al. The 10th PCTL limit was 263 pmol/L (71.6 pg/mL) at Day −4. In this case, to include the 90th PCTL as well as the 10th PCTL, abstinence should begin on ~Day −9 to avoid pregnancy. **Bottom**: The day-specific mean and 5–95th PCTLs for E2 are shown for Stricker et al. The 5th PCTL was 186 pmol/L (50.6 pg/mL) at Day −4. In this case, to include the 95th PCTL as well as the 5th PCTL, abstinence should begin ~Day −14 to avoid pregnancy. The Dighe et al., high-resolution E2 reference ranges had the least variance and therefore the least restrictive FSD for abstinence with a potential FAM of family planning.

This question was investigated by examining the day-specific serum E2 reference levels from three separate laboratories: Dighe et al., Roos et al., and Stricker et al. [18,19,20] and Supplementary Materials, Table S1a–c. The day-specific serum E2 data were plotted with variances as shown in Figure 2. Assuming the interval, Day −4 to Day +1, as the putative day of potential fertility [5,6,7], the lower limit of serum E2 on Day −4 is used to establish a E2 level—a ‘FSD E2 level’—for abstinence to avoid pregnancy with a potential FAM. Consequently, the FSD using the ‘FSD E2 level’ is set for inclusion of upper and lower limit day-specific E2 cohorts. With the Dighe et al., data, there is a FSD E2 level of ~411 pmol/L (112 pg/mL) at Day −4, and a FSD of Day −6. In contrast, the Roos et al., and Stricker et al., data showed FSD E2 levels (Day −4 lower limit E2 levels) of 263 pmol/L (71.6 pg/mL) and 186 pmol/L (50.6 pg/mL), respectively, and because of greater variance in the day-specific serum E2 levels, FSD results of Day −9 and >Day −9, respectively.

In summary, a serum E2 level of ~411 pmol/L (112 pg/mL) could possibly serve as a level for the FSD, or for days with E2 levels <411 pmol/L (112 pg/mL) as ‘safe days’, with FAMs of family planning, but this is highly dependent on the sensitivity and precision of the assay.

### 3.2. A Fertility Indicator Equation Using Serum E2 Levels Maps the Ovulatory Cycle

A better method than simply an isolated serum E2 level was needed for the timing of the fertile phase. To this end, a fertility indicator equation, FIE, was discovered after review of the Dighe et al., Roos et al., and Stricker et al., day-specific serum E2 levels. This formulation with a product of two sequential (normalized) changes in E2 levels and a sign indicating confidence in direction of change (+, −, indeterminate) was found to be powerful in identifying the fertile phase and subsequent transition into the postovulatory phase.

**Figure 3:**
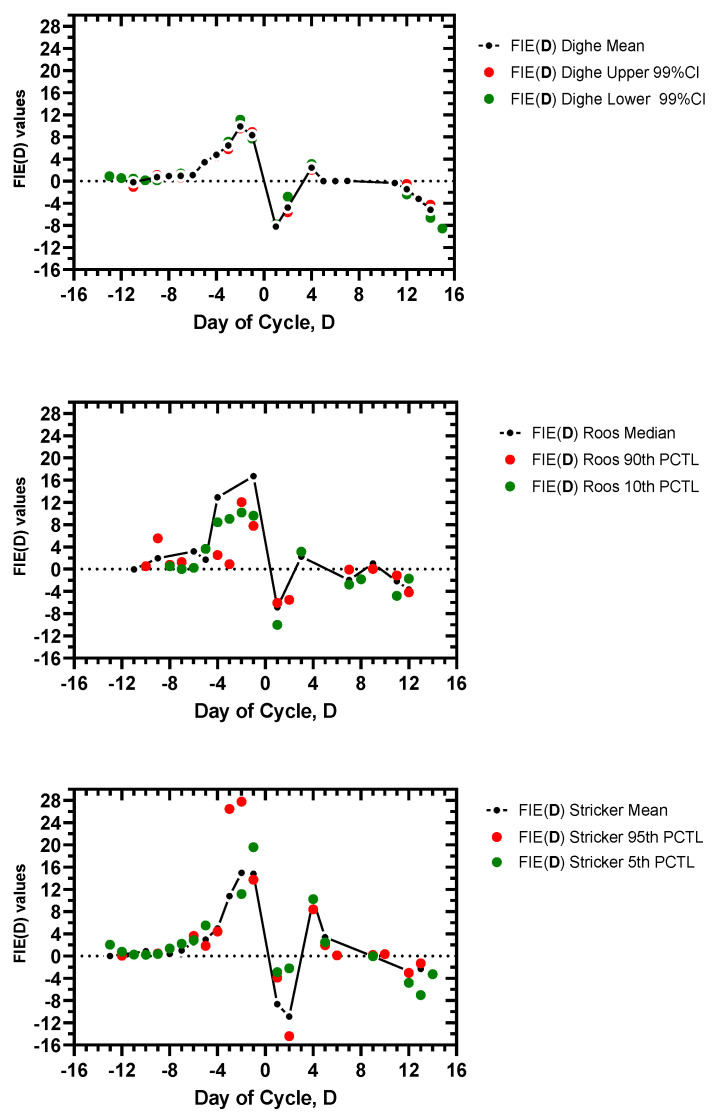
Plot of FIE values, FIE(**D**), for days of cycle, **D**, using the day-specific E2 levels (mean or median, 99%CI, 5–10th PCTL, and 90–95th PCTL limits) from Dighe et al. [18], Roos et al. [19], and Stricker et al. [20]. The formulation for FIE is detailed in Figure 1 and sign can be +, −, or indeterminate. Indeterminate FIE values cannot be plotted, only FIE values with + or − sign. Day 0 is day of peak serum LH.

Table 1 shows the FIE values for day of cycle, FIE(**D**), using the day-specific E2 levels from Dighe et al., days −15 to +7. FIE (**D**) values using the day-specific E2 levels during the entire cycle from Dighe et al., Roos et al., and Stricker et al., are provided in the Supplementary Materials Table S1a–c. FIE(**D**) values for the entire sets of data from Dighe et al., Roos et al., and Stricker et al., are plotted in Figure 3. Only +FIE and −FIE values could be plotted, not ‘ind’ (indeterminate) values.

The FIE (**D**) plots reveal a striking signature, a reproducible pattern for all the day-specific serum E2 sets of data: mean or median, lower 99% CI/10th PCTL/5th PCTL, and upper 99% CI/90th PCTL/95th PCTL for the Dighe et al., Roos et al., and Stricker et al., data. That is, the FIE signature is reproducible over 9 total sets of daily E2 levels. There is: (1) a significant increase in FIE at ~Day −4 or shortly before, (2) a marked maximum FIE after Day −4 and before Day 0, (3) an inflection point with change in sign from +FIE to −FIE at Day 0 (Day 0, indFIE sign), (4) and a short-lived, minor peak in FIE in the luteal phase at ~Day +3 or Day +4.

A suitable FSD using the FIE values could be identified in each data set. A threshold FIE value of ≥2.5 was assigned to best fit the data for a FSD of Day −5 or Day −6; this was the case with the exception of one outlier, Day −4, for the Roos 90th PCTL data (Table 2). An additional rule is required to validate the FSD; a FSD must be confirmed by sequential +FIE values for at least 2 days with at least one value above the 2.5 threshold. This enabled a preovulatory FSD to be determined for each cycle and avoided a discrepancy with the minor postovulatory E2 peak.

Other parameters of the FIE signature are the maximum FIE value and the day of the maximum value, FIE (**MaxDay**) and **MaxDay**, respectively. The early preovulatory phase FIE values were mainly indeterminate sign or had magnitude <1, reflecting little rate of change and background noise. As ovulation approached, +FIE magnitude increased significantly, reaching a peak value of 9.5–27.8, with FIE values of 9.9–16.7 for the mean/median cycles (Table 2). The **MaxDay**s were −2 in 7/9 data sets and −1 in 2/9 data sets (Table 2). Thus, with a FAM of family planning a FIE value of ~9 or higher could signify a maximum time for fertility.

Finally, the FIE signature had a highly reproducible day of transition from the preovulatory to the postovulatory phase. The day of transition was indicated by an indeterminate FIE sign on Day 0, the day of the serum LH peak, with a total change in FIE value between Day −1 and Day +1 that ranged 14–23 (Figure 3, Table 2). Thus, the FIE signature can be used to indicate the transition to the luteal, infertile phase for a FAM of family planning.

## 4. Discussion

The fertility indicator equation developed herein is based upon a product of successive proportional changes relative to the previous day with a sign for confidence in direction. It creates values which oscillate around zero such that trends are removed, but with large jumps indicating something interesting is going-on, such as impending ovulation. Basically, the FIE(**D**) calculations can be done with pencil, paper, and a simple calculator; three initial E2 levels are needed for the first FIE value, then a FIE value is calculated daily based upon the serum (or possibly whole blood) E2 level. We suspect the FIE can also be used with daily urinary E3G data as well and hope for this study in the future.

The use of serum E2 levels for threshold limits in identifying the FSD was considered. The assay results from Dighe et al., had sufficient sensitivity and precision to indicate a serum E2 level of ~411 pmol/L (112 pg/mL) was indicative of the transition to the fertile phase during the ovulatory menstrual cycle, which would establish Day −6 as the day to begin abstinence to avoid pregnancy. However, assay results from the other laboratories had such variance as to make a serum E2 threshold impractical, at least as an indicator for the FSD. Nonetheless, our analysis of the published data suggests that with the sensitivity and precision of Dighe et al., a functional sensitivity of at least 110 pmol/L (30 pg/mL) and a CV of less than 20%, serum and future fingerstick E2 measurements could establish the FSD.

The power of the FIE to determine FSD is that it does not depend upon a threshold E2 level. The FIE is able to ‘catch’ the change in follicle development manifested in a time interval of ~5 days of increase in E2 levels [14]. The FSD is not only set by a corresponding FIE magnitude (≥2.5), but is also validated by at least two consecutive days of +FIE sign with at least one subsequent FIE value of higher magnitude. A good test for the uniqueness of the FSD during an ovulatory cycle was to carry-out the FIE(**D**) computations for the luteal phase. The secondary, smaller E2 luteal peak was captured by the FIE analysis, but the spike in post-ovulatory FIE(**D**) was transient (i.e., no increasing FIE(**D**)) and usually of smaller magnitude than the FIE seen at peak fertility on Day −1 or Day −2.

One limitation of this study is that it used cohorts of ovulatory cycles rather than individual cycles. Since serum E2 data on individual cycles is lacking, we attempted to address this issue by submitting E2 data from three separate laboratories including outlier E2 levels to FIE computations. However, it is recognized that the power of the FIE will need to be tested with individual cycles, using both serum E2 and urinary E3G daily levels.

The FIE with daily serum/fingerstick E2 levels—or possibly daily urinary E3G levels—is not envisioned as a stand-alone technology for FAM technology. The urine LH and urine P3G tests are already available to consumers and strong indicators of ovulation and the luteal phase [1,2,3,21,22,23]. Subjective observations of cervical-vaginal fluid and possible future mucin (CA125) levels in cervical-vaginal fluid could serve in combination as fertile signals [4,21,22]. The FIE would be employed in the preovulatory phase and could be particularly useful in cycles where there is a prolonged phase of follicular development to ovulation. However as shown, the FIE signature can also strikingly signal the transition to luteal phase with a high +FIE (on Day −1) to indFIE (on Day 0) to low −FIE (on Day +1).

Although the FIE was tested in the post-luteal phase where a secondary E2 peak occurs, which informed rules for its usage in fertile phase assessment, it will need to be more rigorously tested with irregular cycles including cycles from patients with polycystic ovary syndrome (PCOS). It is believed that the FIE is particularly suited for PCOS cycles given extended intervals of anovulation. And although outside the scope of this work, we look forward to application of the FIE with urinary E3G data.

## 5. Conclusions

The fertility indicator equation, FIE, using day-specific serum E2 data from ovulatory cycles, has a characteristic signature which maps to the particular phases of the cycle. It can be used to establish the fertile start day (FSD) and signal the transition from ovulation to the luteal phase. It is hoped that the FIE can be used with fingerstick E2 measurements or urinary E3G measurements to improve fertility assessment methods of family planning.

## Figures and Tables

**Table 1:** Delta and fertility indicator equation (FIE) values for day of cycle, **D**, from Dighe et al., data.

Day of Cycle	^a^ Mean E2 Dighe (pg/mL)	Mean E2 Dighe (pmol/L)	^b^ Delta (D) Dighe Mean	^c^ FIE (D) Dighe Mean	E2 Upper 99% Dighe (pmol/L)	Delta (D) Dighe Upper 99%	FIE (D) Dighe Upper 99%	E2 Lower 99% Dighe (pmol/L)	Delta (D) Dighe Lower 99%	FIE (D) Dighe Lower 99%
−15	54.9	202			275			128		
−14	53.9	198	−0.02		253	−0.080		143	0.117	
−13	65	239	0.207	ind0.41	319	0.261	ind2.087	154	0.077	0.901
−12	57.9	213	−0.109	ind2.253	261	−0.182	ind4.743	165	0.071	0.549
−11	56.8	209	−0.019	0.204	246	−0.057	−1.045	176	0.067	0.476
−10	62	228	0.091	ind0.171	275	0.118	ind0.678	180	0.023	0.152
−9	66.9	246	0.079	0.718	301	0.095	1.115	191	0.061	0.139
−8	74.8	275	0.118	0.931	330	0.096	0.911	224	0.173	1.056
−7	80.8	297	0.08	0.943	352	0.067	0.642	242	0.080	1.388
−6	91.9	338	0.138	1.104	400	0.136	0.909	279	0.153	1.229
−5	114.8	422	0.249	3.431	503	0.258	3.511	341	0.222	3.398
−4	136.8	503	0.192	4.77	591	0.175	4.505	411	0.205	4.562
−3	182.8	672	0.336	6.449	786	0.330	5.772	554	0.348	7.142
−2	236.6	870	0.295	9.9	1013	0.289	9.529	731	0.319	11.116
−1	303.6	1116	0.283	8.331	1325	0.308	8.895	907	0.241	7.692
0	251.6	925	−0.171	ind4.839	1068	−0.194	ind5.974	778	−0.142	ind3.424
1	130.8	481	−0.48	−8.215	613	−0.426	−8.263	352	−0.548	−7.788
2	117.8	433	−0.1	−4.79	532	−0.132	−5.629	334	−0.051	−2.800
3	133.8	492	0.136	ind1.36	595	0.118	ind1.565	385	0.153	ind0.781
4	157.8	580	0.179	2.437	697	0.171	2.030	463	0.203	3.094
5	157.8	580	0	0	701	0.006	0.098	459	−0.009	ind0.175
6	161.8	595	0.026	0	716	0.021	0.012	474	0.033	ind0.028
7	164.8	606	0.018	0.048	720	0.006	0.012	496	0.046	0.152

^a^ Day-specific mean, upper 99% CI limit, lower 99% CI limit reference range serum E2 levels from Dighe et al. [18]. Days of the cycle are indexed to. Day 0, day of serum LH peak. ^b^ Delta (**D**) is calculated for day, **D** by: [E2 on day, **D** − E2 on day, **D-1**]/E2 on day, **D-1** and for day, **D-1** by: [E2 on Day, **D-1** − E2 on day, **D-2**]/E2 on day, **D-2**. ^c^ The magnitude of FIE on day, **D**, is FIE(**D**) and is calculated by: ((Delta(**D**) × (Delta(**D-1**) × 100). The sign of FIE(**D**) is +, −, or indeterminate (written as in d) and is assigned as follows: +Delta(**D**) × +Delta(**D-1**) is +; −Delta(**D**) × −Delta(**D-1**) is −; and −Delta(**D**) × +Delta(**D-1**) or +Delta(**D**) × −Delta(**D-1**) is ind.

**Table 2:** Fertile start day, FSD, and day of maximum FIE value, MaxDay, corresponding FIE values, FIE (FSD) and FIE (MaxDay), and day of significant change in magnitude and sign (+ to −) of FIE, the transition day.

^a^ Dighe, Roos, Stricker Cycles	^b^ FSD	FIE (FSD)	MaxDay	FIE (MaxDay)	^c^ Transition Day
Dighe Mean cycles	−5	3.4	−2	9.9	0
Dighe Upper 99% cycles	−5	3.5	−2	9.5	0
Dighe Lower 99% cycles	−5	3.4	−2	11.1	0
Roos Median cycles	−6	3.2	−1	16.7	0
Roos 90th PCTL cycles	−4	2.5	−2	12	0
Roos 10th PCTL cycles	−5	3.6	−2	10.2	0
Stricker Mean cycles	−6	2.5	−2	15	0
Stricker 95th PCTL cycles	−6	3.6	−2	27.8	0
Stricker 5th PCTL cycles	−6	2.9	−1	19.6	0

^a^ Day-specific serum E2 levels with number of subjects and statistics are provided by Dighe et al. [18], Roos et al. [19], and Stricker et al. [20]. Their cycles are indexed to the serum LH peak (Day 0). ^b^ For calculation of the FIE values see Methods and Table 1. FSD is the first day of the cycle where FIE is ≥2.5 and validated by sequential increase rule. ^c^ The transition day is the day of indeterminate sign bracketed by change in sign from + to − with magnitude change >15.

## Supplementary Materials

The following are available online at https://www.mdpi.com/1010-660X/56/11/555/s1, Table S1a–c. Complete delta and fertility indicator equation (FIE) values for day of cycle, **D**, from Dighe et al., Roos et al., and Stricker et al.
