# Supplementary material for: A Novel Fertility Indicator Equation Using Estradiol Levels for Assessment of Phase of the Menstrual Cycle
Source: Medicina (Kaunas). 2020 Oct 22;56(11):555. doi: 10.3390/medicina56110555 (PMC7690440; doi:10.3390/medicina56110555)
Supplement: Supplementary file 1 [file medicina-56-00555-s001.pdf]

SUPPLEMENTAL TABLES: COMPLETE DIGHE, ROOS, STRICKER COMPUTATIONS

Table 1a. Delta and Fertility Indicator Equation (FIE) values for day of cycle, **D**, from Dighe et al. data (Day -15 to Day 15).

Day of Cycle	[a]Mean E2 Dighe (pg/ml)	Mean E2 Dighe (pmol/L)	[b]Delta(**D**) Dighe Mean	[c]FIE(**D**) Dighe Mean	E2 Upper 99%CI Dighe (pmol/L)	Delta(**D**) Dighe Upper 99%	FIE(**D**) Dighe Upper 99%	E2 Lower 99%CI Dighe (pmol/L)	Delta(**D**) Dighe Lower 99%	FIE(**D**) Dighe Lower 99%
-15	54.9	202			275			128		
-14	53.9	198	-0.02		253	-0.08		143	0.117	
-13	65	239	0.207	ind0.41	319	0.261	ind2.087	154	0.077	0.901
-12	57.9	213	-0.109	ind2.253	261	-0.182	ind4.743	165	0.071	0.549
-11	56.8	209	-0.019	-0.204	246	-0.057	-1.045	176	0.067	0.476
-10	62	228	0.091	ind0.171	275	0.118	ind0.678	180	0.023	0.152
-9	66.9	246	0.079	0.718	301	0.095	1.115	191	0.061	0.139
-8	74.8	275	0.118	0.931	330	0.096	0.911	224	0.173	1.056
-7	80.8	297	0.08	0.943	352	0.067	0.642	242	0.08	1.388
-6	91.9	338	0.138	1.104	400	0.136	0.909	279	0.153	1.229
-5	114.8	422	0.249	3.431	503	0.258	3.511	341	0.222	3.398
-4	136.8	503	0.192	4.77	591	0.175	4.505	411	0.205	4.562
-3	182.8	672	0.336	6.449	786	0.33	5.772	554	0.348	7.142
-2	236.6	870	0.295	9.9	1013	0.289	9.529	731	0.319	11.116
-1	303.6	1116	0.283	8.331	1325	0.308	8.895	907	0.241	7.692
0	251.6	925	-0.171	ind4.839	1068	-0.194	ind5.974	778	-0.142	ind3.424
1	130.8	481	-0.48	-8.215	613	-0.426	-8.263	352	-0.548	-7.788
2	117.8	433	-0.1	-4.79	532	-0.132	-5.629	334	-0.051	-2.8
3	133.8	492	0.136	ind1.36	595	0.118	ind1.565	385	0.153	ind0.781
4	157.8	580	0.179	2.437	697	0.171	2.03	463	0.203	3.094
5	157.8	580	0	0	701	0.006	0.098	459	-0.009	ind0.175

6	161.8	595	0.026	0	716	0.021	0.012	474	0.033	ind0.028
7	164.8	606	0.018	0.048	720	0.006	0.012	496	0.046	0.152
8	148.8	547	-0.097	ind0.18	650	-0.097	ind0.054	444	-0.105	ind0.487
9	163.7	602	0.101	ind0.979	727	0.118	ind1.152	477	0.074	ind0.779
10	157.8	580	-0.037	ind0.367	683	-0.061	ind0.717	474	-0.006	ind0.047
11	142.8	525	-0.095	-0.347	661	-0.032	-0.195	393	-0.171	-0.107
12	120.8	444	-0.154	-1.463	554	-0.162	-0.521	338	-0.14	-2.392
13	95.7	352	-0.207	-3.197	437	-0.211	-3.419	264	-0.219	-3.064
14	71.8	264	-0.25	-5.18	349	-0.201	-4.253	184	-0.303	-6.634
15	74.8	275	0.042	ind1.042	422	0.209	ind4.212	132	-0.283	-8.564

[a]Day-specific mean, upper 99% CI limit, and lower 99% CI limit reference range serum E2 levels from Dighe et al. [17].
[b]Delta(**D**) is calculated for day, **D** by: [E2 on day, **D** – E2 on day, **D-1**]/E2 on day**, D-1**.
[c]The magnitude of FIE on day, **D**, is FIE(**D**) and is calculated by: ((Delta(**D**) X (Delta(**D-1**) * 100). The sign of FIE(**D**) is +, -, or indeterminate (written as ind) and is assigned as follows: +Delta(**D**) X +Delta(**D-1**) is +;
-Delta(**D**) X -Delta(**D-1**) is -; and -Delta(**D**) X +Delta(**D-1**) or +Delta(**D**) X -Delta(**D-1**) is ind.

Table 1b. Delta and Fertility Indicator Equation (FIE) values for day of cycle, **D**, from Roos et al. data (Day -15 to Day 12).

Day of Cycle	[a]Median E2 Roos (pg/ml)	Median E2 Roos (pmol/L)	[b]Delta(**D**) Roos Median	[c]FIE(**D**) Roos Median	E2 90th PCTL Roos (pmol/L)	Delta(**D**) Roos 90th PCTL	FIE(**D**) Roos 90th PCTL	E2 10th PCTL Roos (pmol/L)	Delta(**D**) Roos 10th PCTL	FIE(**D**) Roos 10th PCTL
-15	44.0	161.9			177.5			106.7		
-14	22.8	83.7	-0.483		83.7	-0.528		83.7	-0.216	
-13	44.8	164.8	0.969	ind46.801	166.3	0.987	ind52.151	156.3	0.867	ind18.697
-12	42.0	154.5	-0.063	ind6.056	165.2	-0.007	ind0.653	128	-0.181	ind15.705
-11	41.6	153.1	-0.009	-0.057	168.1	0.018	ind0.012	143.4	0.120	ind2.178
-10	44.1	162.1	0.059	ind0.053	218.1	0.297	0.522	81.9	-0.429	ind5.160
-9	58.7	215.9	0.332	1.951	258.8	0.187	5.551	135.1	0.650	ind27.858
-8	47.6	175.1	-0.189	ind6.272	269.5	0.041	0.772	136.2	0.008	0.529
-7	64.9	238.6	0.363	ind6.853	351.9	0.306	1.264	138.5	0.017	0.014
-6	70.6	259.6	0.088	3.192	334.6	-0.049	ind1.503	157.6	0.138	0.233

-5	84.3	309.8	0.193	1.702	608.1	0.817	ind4.018	199.3	0.265	3.649
-4	140.4	516.3	0.667	12.890	626.9	0.031	2.527	262.8	0.319	8.430
-3	121.6	446.9	-0.134	ind8.960	809.1	0.291	0.899	337.3	0.283	9.032
-2	212.1	779.7	0.745	ind10.010	1144.5	0.415	12.048	458.4	0.359	10.178
-1	259.7	954.6	0.224	16.705	1359.4	0.188	7.784	580.9	0.267	9.594
0	232.0	853.1	-0.106	ind2.385	1220.8	-0.102	ind1.914	489.3	-0.158	ind4.214
1	82.0	301.6	-0.646	-6.874	494.1	-0.595	-6.069	177.9	-0.636	-10.035
2	85.6	314.6	0.043	ind2.786	448.2	-0.093	-5.530	248.3	0.396	ind25.185
3	130.2	478.7	0.522	2.248	595.1	0.328	ind3.045	268	0.079	3.140
4	72.4	266.1	-0.444	ind23.166	266.1	-0.553	ind18.120	266.1	-0.007	ind0.056
5	196.0	720.6	1.708	ind75.856	720.6	1.708	ind94.427	720.6	1.708	ind1.211
6	186.9	687.2	-0.046	ind7.917	687.2	-0.046	ind7.917	687.2	-0.046	ind7.917
7	107.3	394.6	-0.426	-1.974	675.8	-0.017	-0.077	274.4	-0.601	-2.784
8	111.3	409.3	0.037	ind1.586	681.5	0.008	ind0.014	266.1	-0.030	-1.817
9	141.7	521.1	0.273	1.018	698.9	0.026	0.022	355.1	0.334	ind1.012
10	125.6	461.8	-0.114	ind3.108	672.9	-0.037	ind0.095	286	-0.195	ind6.508
11	101.5	373.3	-0.192	-2.181	468.1	-0.304	-1.132	215.3	-0.247	-4.810
12	82.2	302.1	-0.191	-3.655	403.7	-0.138	-4.187	200.5	-0.069	-1.699

[a]Day-specific median, 90th percentile (PCTL), and 10th PCTL reference range serum E2 levels from Roos et al. [18].
[b]Delta(**D**) is calculated as in table 1a.
[c]The magnitude and sign of FIE(**D**) and is in table 1a.

Table 1c. Delta and Fertility Indicator Equation (FIE) values for day of cycle, **D**, from Stricker et al. data (Day -15 to Day 14).

Day of Cycle	Mean E2 Stricker (pg/ml)	Mean E2 Stricker (pmol/L)	Delta(**D**) Stricker Mean	FIE(**D**) Stricker Mean	E2 95th PCTL Stricker (pmol/L)	Delta(**D**) Stricker 95th PCTL	FIE(**D**) Stricker 95th PCTL	E2 5th PCTL Stricker (pmol/L)	Delta(**D**) Stricker 5th PCTL	FIE(**D**) Stricker 5th PCTL
-15	32.21	118.42			194.44			57.55		
-14	36.18	133.01	0.123		187.67	-0.035		66.06	0.148	

-13	36.22	133.17	0.001	0.015	189.34	0.009	ind0.031	75.2	0.138	2.046
-12	34.26	125.95	-0.054	ind0.007	200.97	0.061	0.055	79.42	0.056	0.776
-11	36.62	134.65	0.069	ind0.375	196.75	-0.021	ind0.129	82.98	0.045	0.252
-10	41.16	151.33	0.124	0.856	209.56	0.065	ind0.137	87.6	0.056	0.250
-9	43.38	159.48	0.054	0.667	224.68	0.072	0.470	93.4	0.066	0.369
-8	46.33	170.34	0.068	0.367	220.86	-0.017	ind0.123	112.49	0.204	1.353
-7	53.28	195.9	0.150	1.022	267.33	0.210	ind0.358	124.67	0.108	2.213
-6	62.07	228.2	0.165	2.474	313.36	0.172	3.623	157.66	0.265	2.865
-5	73.19	269.07	0.179	2.953	346.76	0.107	1.835	190.47	0.208	5.507
-4	93.48	343.68	0.277	4.966	490.81	0.415	4.428	186.47	-0.021	ind0.436
-3	129.82	477.27	0.389	10.778	803.56	0.637	26.471	241.52	0.295	ind0.619
-2	179.84	661.19	0.385	14.979	1153.76	0.436	27.770	337.35	0.397	11.168
-1	248.84	914.84	0.384	14.783	1517.67	0.315	13.746	503.85	0.494	19.583
0	212.37	780.76	-0.147	ind5.622	1425.39	-0.061	ind1.918	482	-0.043	ind2.140
1	87.20	320.59	-0.589	-8.638	514.35	-0.639	-3.886	159.87	-0.668	-2.898
2	71.08	261.32	-0.185	-10.896	398.38	-0.225	-14.411	154.62	-0.033	-2.195
3	92.19	338.94	0.297	ind5.491	495.82	0.245	ind5.515	208.64	0.349	ind1.147
4	123.51	454.07	0.340	10.089	665.99	0.343	8.395	269.8	0.293	10.241
5	135.86	499.49	0.100	3.398	703.85	0.057	1.951	292.57	0.084	2.474
6	135.20	497.07	-0.005	ind0.048	719.72	0.023	0.128	267.07	-0.087	ind0.736
7	144.47	531.14	0.069	ind0.033	695.47	-0.034	ind0.076	291.71	0.092	ind0.804
8	137.19	504.39	-0.050	ind0.345	718.2	0.033	ind0.110	281.91	-0.034	ind0.310
9	135.78	499.18	-0.010	-0.052	764.41	0.064	0.210	279.18	-0.010	-0.033
10	143.26	526.68	0.055	ind0.057	806.7	0.055	0.356	294.48	0.055	ind0.053
11	95.38	350.65	-0.334	ind1.841	640.16	-0.206	ind1.142	210.22	-0.286	ind1.568
12	87.65	322.24	-0.081	-2.708	546.15	-0.147	-3.032	174.82	-0.168	-4.818
13	62.48	229.7	-0.287	-2.327	497.74	-0.089	-1.302	101.93	-0.417	-7.021
14	67.80	249.28	0.085	ind2.448	693.54	0.393	ind3.487	93.97	-0.078	-3.256

[a]Day-specific mean, 95th percentile (PCTL), and 5th PCTL reference range serum E2 levels from Stricker et al. [19].

[b]Delta(**D**) is calculated as in table 1a.

[c]The magnitude and sign of FIE(**D**) and is in table 1a.
